# Mapping risk of ischemic heart disease using machine learning in a Brazilian state

**DOI:** 10.1371/journal.pone.0243558

**Published:** 2020-12-10

**Authors:** Marcela Bergamini, Pedro Henrique Iora, Thiago Augusto Hernandes Rocha, Yolande Pokam Tchuisseu, Amanda de Carvalho Dutra, João Felipe Herman Costa Scheidt, Oscar Kenji Nihei, Maria Dalva de Barros Carvalho, Catherine Ann Staton, João Ricardo Nickenig Vissoci, Luciano de Andrade

**Affiliations:** 1 Postgraduate Program in Health Sciences, State University of Maringa, Maringa, Brazil; 2 Department of Medicine, State University of Maringa, Maringa, Brazil; 3 Duke Global Health Institute, Duke University, Durham, North Carolina, United States of America; 4 Education, Letters and Health Center, State University of the West of Parana, Foz do Iguaçu, Parana, Brazil; 5 Division of Emergency Medicine, Department of Surgery, Duke University Medical Center, Durham, North Carolina, United States of America; University of Minnesota, UNITED STATES

## Abstract

Cardiovascular diseases are the leading cause of deaths globally. Machine learning studies predicting mortality rates for ischemic heart disease (IHD) at the municipal level are very limited. The goal of this paper was to create and validate a Heart Health Care Index (HHCI) to predict risk of IHD based on location and risk factors. Secondary data, geographical information system (GIS) and machine learning were used to validate the HHCI and stratify the IHD municipality risk in the state of Paraná. A positive spatial autocorrelation was found (Moran’s I = 0.6472, p-value = 0.001), showing clusters of high IHD mortality. The Support Vector Machine, which had an RMSE of 0.789 and error proportion close to one (0.867), was the best for prediction among eight machine learning algorithms after validation. In the north and northwest regions of the state, HHCI was low and mortality clusters patterns were high. By creating an HHCI through ML, we can predict IHD mortality rate at municipal level, identifying predictive characteristics that impact health conditions of these localities’ guided health management decisions for improvements for IHD within the emergency care network in the state of Paraná.

## Introduction

Cardiovascular diseases (CVDs) are the leading cause of deaths globally. Among all cardiovascular diseases, ischemic heart disease (IHD) causes the most deaths, both in high-income countries as well as in low- and middle-income countries. More than nine million people died from IHD in 2016 worldwide [1]. Brazil had an average of 306 IHD deaths daily, or one death every five minutes [2]. Cardiac diagnostic tests availability are limited, mostly occurring in urban centers [3], disproportionately limiting the accessibility of remote population and those from small cities.

A vast amount of research on risk factors predicts negative outcomes for patients with IHD. Most studies addressing the prediction of IHD mortality are related to the individual after the event and use secondary data collected from medical records [4,5]. However, models based on individual data do not represent cultural and socioeconomic disease determinants, which are essential for public health policy and population well-being. The relationship between municipality indicators with socioeconomic and demographic factors, health coverage, and high mortality IHD rates is well documented in the scientific literature [6–8].

Traditional methodological approaches using linear models are usually unable to show subtle associations between indicators and mortality rates or provide insights into factors that can affect health management [9–12]. However, few methodological validated studies seek to predict IHD mortality rates using a data-based innovation that stratifies municipal IHD mortality risk as a public health planning strategy. No machine learning studies predict mortality IHD rate using municipal data.

Using machine learning to generate a risk score allows the use of a greater number of variables at the same time, achieving greater sensitivity and specificity and providing robust results [5,13–15]. Machine learning is better than calculating an index from basic mathematical functions with indicators chosen by experts because it reduces methodological bias risks, enables the discovery of previously unknown regularities and is more reliable in decision-making [8,16–18].

The objective of this study was to create and validate the Heart Health Care Index (HHCI), using a machine-learning algorithm to stratify the IHD municipal risk in the state of Paraná, Brazil. With this index, it will be possible to propose actions and targets to reduce regional disparities. This tool also serves as a source of information for the redesign of actions in IHD care within the emergency care network in the Brazilian state of Paraná.

## Materials and methods

### Study design and location

This is an observational, cross-sectional, ecological study that used secondary data, spatial analysis and machine learning techniques to create and validate the Heart Health Care Index (HHCI). Once validated, the HHCI was used to stratify the municipal risk for IHD from people who are 20 to 79 years of age, in the 399 municipalities of Paraná state.

Paraná has more than 11.34 million inhabitants (5.44% of the Brazilian population) and is located in the southern region of the country. Municipalities are distributed into 10 administrative regions, with about 90% of them having fewer than 50,000 inhabitants [19]. Paraná ranks among the top five in Human Development Index (HDI: 0.749) among all the 27 Brazilian federative units, being classified as high HDI [20].

This study was approved by the Ethics Committee of State University of Maringá under no. 2.232.580 and was exempted from informed consent.

### Source data and study variables

Population, socioeconomic and demographic information as well as the georeferenced cartographic base of Paraná state were obtained from the Brazilian Institute of Geography and Statistics (IBGE) [19]. Information of health coverage was obtained from Ambulatory Information System Database (SIA/DATASUS) [http://www2.datasus.gov.br/DATASUS/index.php?area=0202&id=19122] and IHD mortality was obtained from the Mortality Information System of the Ministry of Health (SIM/DATASUS) [http://www2.datasus.gov.br/DATASUS/index.php?area=0205&id=6937] and expressed in relative and absolute values. In this study, the variables were collected at the municipality level. Our analysis compared rates of IHD by municipalities, seeking to fit a prediction model from municipality level socioeconomic, demographic and health coverage information.

#### IHD mortality

We selected IHD cases using the International Statistical Classification of Diseases and Related Health Problems–10th Revision (ICD-10), specifically as codes I20 to I25 [1]. To minimize possible mortality-related data fluctuations, the average mortality rates per 100,000 inhabitants (age-adjusted) for the 2009–2014 period were calculated as the outcome for algorithm learning and the 2015 mortality rate was used for model validation [21]. Mortality rates were smoothed through the Empirical Bayesian Estimator for each municipality in Paraná state using GeoDa™ software [22] to reduce extreme behaviors in different size populations. Predictive variables were composed of socioeconomic, demographic and health coverage indicators for each of the 399 cities.

#### Municipal socioeconomic and demographic indicators

Socioeconomic and demographic: 1. Gross domestic product (GDP); 2. Municipal Human Development Index (MHDI); 3. Expectation of years of child study; 4. Illiteracy rate; 5. Percentage of people with elementary school; 6. GINI index; 7. Average household income per capita, 8. Total employed population; 9. Informal employment rate; 10. Unemployment rate; 11. Income ratio; 12. Percentage of the population with low-income by municipality; 13. Aging rate; 14. Demographic density; 15. Degree of urbanization.

#### Municipal health indicators

Health management conditions: 1. Municipalities were classified in seven strata according to their size and features: small (unfavorable, regular, favorable), medium (unfavorable, regular, favorable) and large, in which similar municipalities were grouped as unfavorable, regular, favorable, based on 14 indicators with four basic categories (demographic, socioeconomic, health and structural service) [23].

Health coverage indicators: The following variables were used: 1. Family health strategy coverage ratio (%); 2. Rates of consultation for diabetic population; 3. Rates of consultation for hypertensive population; imaging tests per 10,000 inhabitants (4. Cardiac catheterization; 5. Myocardial scintigraphy; 6. Echocardiography); 7. IHD morbidity rate per 100000 inhabitants; 8. Cardiologists; 9. Hemodynamic laboratories, ambulances (10. basic and 11. advanced); 12. Spatial accessibility indexes.

A spatial accessibility index, ranging from 0 (poor index) to 1 (ideal index), measured the proximity and availability of sufficient provision of care for the population. This index was generated using the *two-step floating catchment area (2SFCA) method*. This method has two steps, where catchment areas are created by using 60-minute buffers generated by service area (network analysis) from distance and highways speed using software ArcMap (version 10.5). For the first step, catchment areas from the provider to population were created and extension of the proportion calculated from the centroids is verified in the second step [24,25].

### Data analysis

#### Geospatial analysis

First, choropleth maps were generated with IHD smoothed mortality rates divided into quantiles ranges in the municipalities studied. The exploratory spatial data analysis (ESDA) was used to determine the measurements of Global Spatial Autocorrelation (Moran’s I) and Local Indicators of Spatial Association (LISA) using GeoDa software (version 1.12.0, 2017) and QGIS (version 2.14.20).

#### HHCI development

**Fig 1:**
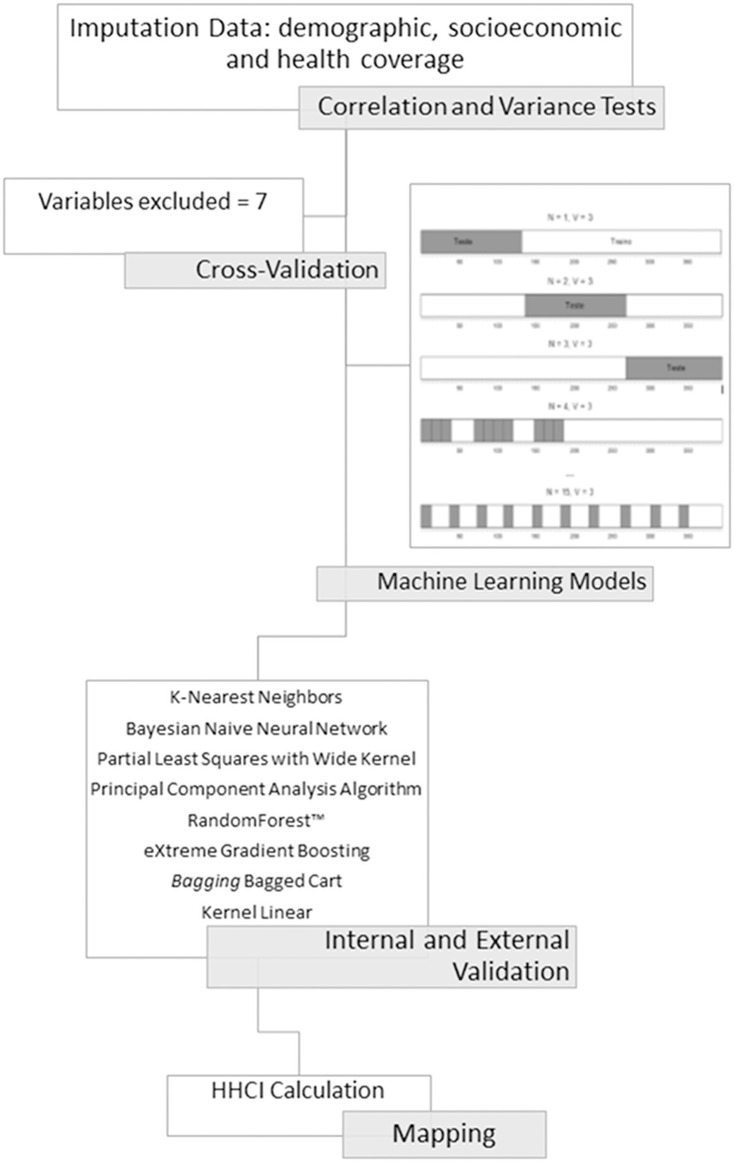
Representative flowchart of the main machine learning model development stages. 1) Variables pre-processing (correlation and variation tests); 2) Predictive variables cross-validation; 3) Machine learning models discrimination and validation (internal and external validation); 4) Heart Health Care Index (HHCI) calculation and mapping.

Machine learning models (Fig 1) were used and constructed according to the TRIPOD guideline [26].

#### Pre-processing

All the variables were initially included in one of the three strata (socioeconomic, demographic or health coverage) with no greater or less pre-hypothesis importance. The caret package [27] had functions integrated in RStudio (version 3.4.4) and was used for training and prediction functions of essential algorithm. The *doSNOW* package was used for parallel multiprocessing. Thirty-seven variables were registered and pre-processed; two identification variables were removed (IBGE codes and city names). The scale native algorithm centralized the Xn numeric variables and these were staggered to X’n. Pearson correlation identified multicollinearity for each of the variables pairs among the 35 remaining indicators (correlation matrix 35x35). A near-zero variance algorithm was applied to the variables, but no significant near-zero variance was found [27].

#### Cross-validation

Cross-validation was performed using predictive variables as a training parameter for the mean of the period 2009–2014. The data (n = 399) was separated into three equal blocks; two were used for the training algorithm and the rest were used for internal validation. This process was repeated 15 times, having a different validation block each time. The outcomes of this process are used to optimize the algorithm trained for independent database future predictions. For each repetition, one of the three blocks were used for validation. The observed mortality rate was just presented to the model for comparison with the predicted one.

#### Models discrimination

Eight machine learning models were tested and compared according to their mortality rate predictive accuracy [28]. The final predictive model was chosen from the following algorithms: K-Nearest Neighbors Algorithm (“knn” in CRAN-R *caret* package v.6.0–80) [27]; Bayes Theorem Algorithms: Bayesian Naive Neural Network (”brnn” in CRAN-R *brnn* package v.0.7) [29]; Dimensional Reduction Algorithms: Partial Least Squares with Wide Kernel Algorithm e Principal Component Regression Algorithm (“kernelpls” e “pcr” in CRAN-R *pls* package v.2.7–0) [30]; Decision Tree Models: RandomForest™ (“rf” in CRAN-R *randomForest* package v.4.6–14 May 2018) [31], por *Bagging* Bagged Cart (“TreeBag” in CRAN-R *caret* package v.6.0–80) [27] e eXtreme Gradient Boosting (“xgbDART” in CRAN-R *xgboost* package v.0.71.2) [32]; Vector Support Machine Algorithm with Kernel Linear (“svmLinear” in CRAN-R *kernlab* package v.0.9–27) [33].

Root Mean Square Error (RMSE) between observed mortality rate and predicted mortality rate was used to evaluate the performance of models for each of the 399 municipalities. The RMSE is calculated according to the formula:

$$\mathrm{RMSE}=\sqrt{\frac{\sum_{i=1}^{n}{\left(P_{i}–O_{i}\right)}^{2}}{n}}$$

where P = predicted mortality rate, O = observed mortality rate, n = number of municipalities. This is a bank/variable dependent metric.

A lower RMSE indicates smaller mean differences between observed mortality rate and predicted mortality rate.

#### Models validation

The RMSE has no reference values because it depends on the dimension of results variables where greater accuracy is represented by smaller RMSE values [34]. However, RMSE usually presents low values when overfitting occurs, which indicates the convergence of the observed mortality rate and predicted mortality rate trending towards 100% approximation [35]. The phenomenon of overfitting can occur when training data are exposed to machine learning models. Overfitting is defined as an exceptional fit to the training data but is inaccurate when predicting unknown values. It is detected when the algorithm prediction training has near-perfect accuracy.

To detect models overfitting and adequacy, two exclusive datasets not included in the training phase validation were created. The 2014 and 2015 mortality rates databases were used as independent datasets to test the trained machine learning algorithm external validity.

The 2014 data are intermediate between the average of the years 2009–2014, unknown to the model when isolated. The 2015 dataset presents values never seen before by the algorithm. The indicators values and 2015 rate follow the trend pattern with 2014 and with the previous years, and thus could be used to validate and adapt the over-adjusted models or non-over-adjusted models.

#### Model calibration

Model calibration was performed comparing the validation on 2015 data to check for RMSE stability and exclude randomly generated results. Additionally, the performed procedure aimed to avoid tuning specific model parameters biases from model to model. The calibration plot was used to assess the predicted versus the actual values regarding each unit of analysis.

#### Heart health care index calculation

Prediction is the secondary goal of HHCI outcome. Through the predicted mortality rate compared to the observed mortality rate, it is possible to define for each municipality an index of health attention.

Each of the models, with a different mathematical logic, generated a complex object of calculation that presented each of the variables associated with a weight coefficient (WC). Thus, predicted mortality rate (PMR) can be expressed and obtained by the formula:

$${\mathrm{PMR}}_{n}=W_{1}C_{1_{n}}+W_{1}C_{1_{n}+\ldots }W_{28}C_{28n}$$

The predicted mortality rate (PMR) of the chosen model is again scaled within the range 0 to 1 and its values are adjusted according to the formula:

$$MR_{\mathrm{adj}(0–1)}=\frac{{\mathrm{MR}}_{P}-\min\left({\mathrm{MR}}_{P}\right)}{\max\left({\mathrm{MR}}_{P}\right)-\min\left({\mathrm{MR}}_{P}\right)}$$

where, min = minimum and max = maximum.

MR_adj(0–1)_ is the complement of the index and represents spatially adjusted and interval-graded smoothed mortality. This value represents a negative outcome, indicating that higher values of MR_adj(0–1)_ represents lower access to health for IHD.

Index (I) is obtained according to:

$$I_{n}=1-{\mathrm{MR}}_{\mathrm{adj}(0–1)n}$$

A choropleth map with spatial distribution of HHCI was generated.

## Results

### IHD mortality in the state of Paraná

IHD deaths numbered 32310 from 2009–2015 in the state of Paraná for a 20–79 year old population, predominantly male (64.2%), with 1 to 3 years of elementary schooling (31.4%), white (78.9%) and married (54.6%).

**Fig 2:**
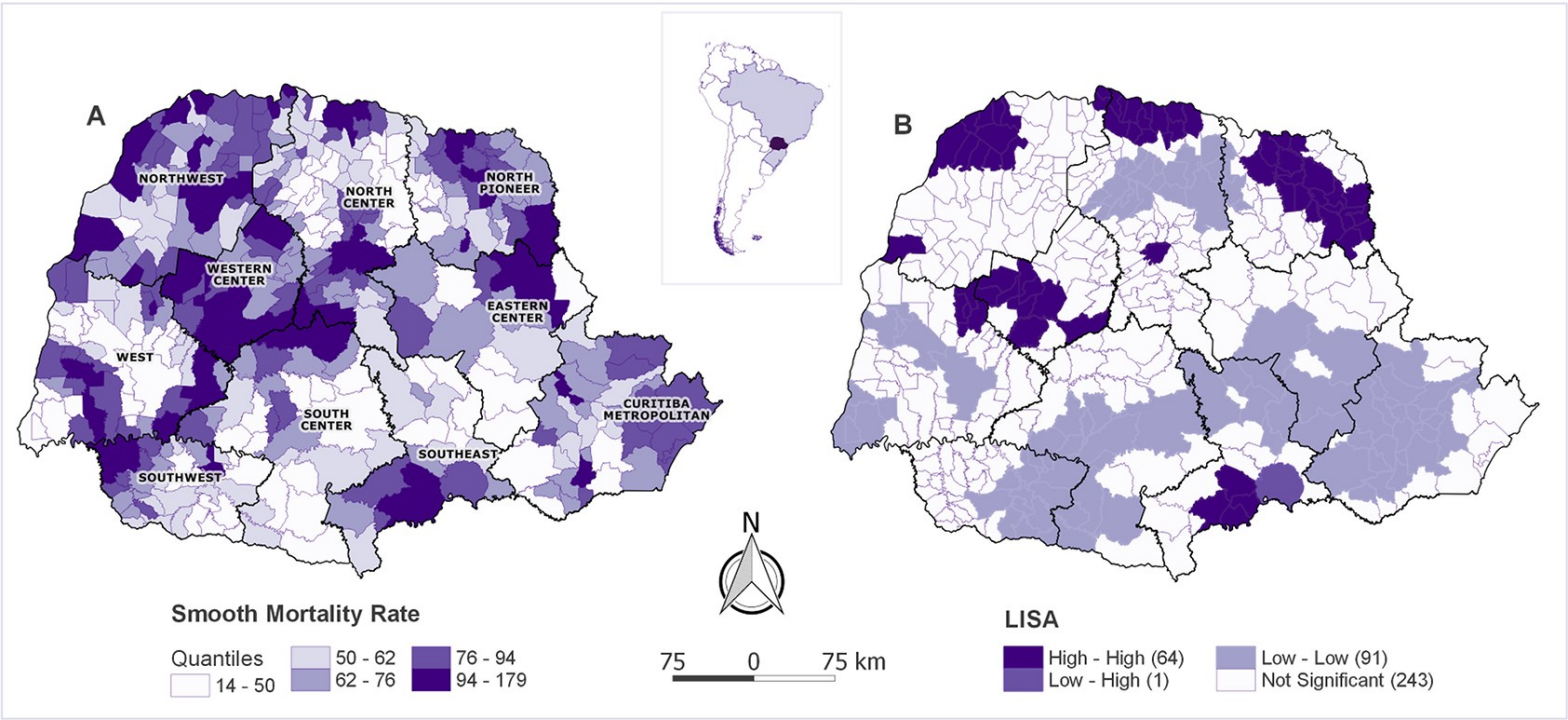
Spatial exploratory analysis of municipalities’ IHD mortality rates. A—Distribution of spatially IHD mortality rates observed in the state of Paraná from 2009–2015 in quantiles by municipality. B—Local Indicators of Spatial Association (LISA) of IHD mortality rates by municipality.

The exploratory analysis of spatial IHD mortality rates (Fig 2A) using the Moran Global and LISA Analysis showed a strong spatial dependence, identifying significant spatial clusters (I = 0.6472, p = 0.001) with high IHD mortality rates municipalities surrounded by high rates municipalities (Fig 2B).

### Pre-processing

All predictive variables were tested for linear correlation using Pearson’s *r* index. The test was performed by generating all possible pairs of 35 variables and calculating the *r* value of each pair. Pairs with an *r* value equal/greater than 0.7 or equal/smaller than -0.7 were found and separated, for a total of 20 [36].

As a result, seven variables were excluded: five socioeconomic (average household income per capita, MHDI, percentage of inhabitants in households with no complete elementary education, occupation rate and income ratio), one demographic (degree of urbanization) and one related to access (index of accessibility to cardiologists) for having higher mean absolute Pearson’s *r* value in all their composed pairs.

### Machine learning models calibration for IHD prediction

The best algorithms presented smaller and more consistent RMSE variability between predicted and observed mortality rates. Data variability was measured using the error proportionality (close to 1) being the larger error the lower proportion index (Table 1).

**Table 1:** Calibration of tested models ordered according to the performance indicator (RMSEs and proportionality between the RMSEs).

MODELS	RMSEs			PROPORTIONALITY	
	2009–2014	2014	2015	2014	2015
XGB	0.299	0.835	0.877	0.358	0.341
RF	0.308	0.832	0.894	0.37	0.345
BRNN	0.491	0.945	1.059	0.52	0.464
Tree Bag	0.551	0.871	0.871	0.633	0.633
**PLS**	**0.771**	**0.912**	**0.932**	**0.845**	**0.827**
**PCR**	**0.786**	**0.917**	**0.919**	**0.855**	**0.855**
**SVM**	**0.790**	**0.910**	**0.940**	**0.867**	**0.840**
KNN	0.842	0.915	0.916	0.92	0.919

BRNN–Bayesian Naive Neural Network; KNN–K-Nearest Neighbors Algorithm; PCR–Principal Component Regression Algorithm; PLS–Partial Least Squares with Wide Kernel Algorithm; RF–Random Forest; SVM–Support Vector Machine; TreeBag; XGB–xbgDART/RMSE–Root Mean Square Error of Prediction.

**Fig 3:**
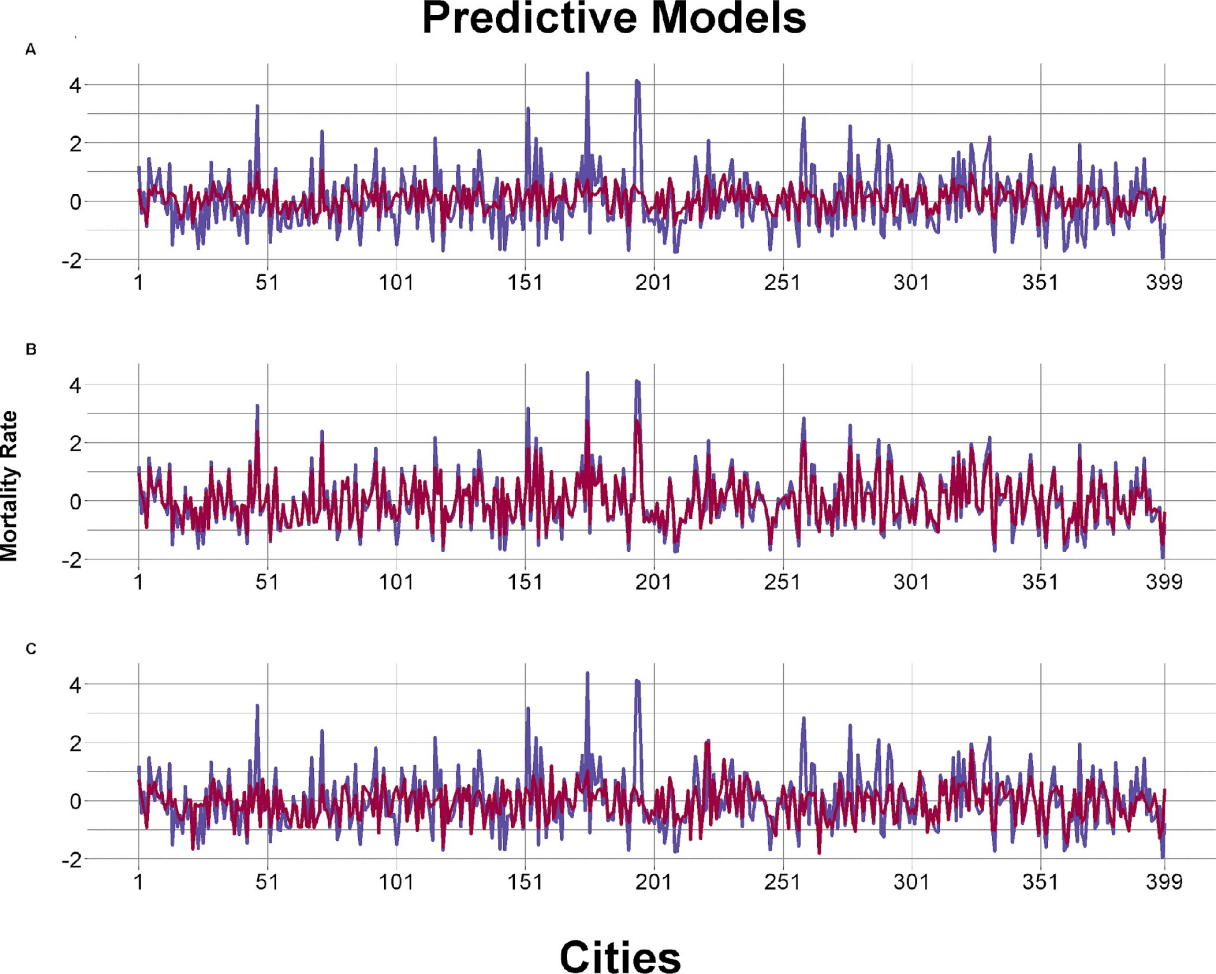
Calibration graphs of the tested predictive models (adjustments using RMSE). A- Example of underfitting calibration model with the worst adjustment (K-Nearest Neighbors); B- Example of overfitting calibration model (Random Forest); C: Example of best fit model (Support Vector Machine). Blue represents the observed mortality rate and red represents the predicted one.

The calibration was evaluated comparing the predicted and the observed values as indicated in the Fig 3, and a suitable adjustment was confirmed between the predicted and the observed outcome variables. Three machine learning models presented more robust results to predict IHD mortality rates for 100000 inhabitants presenting the lowest RMSE values and lower variability during the process: Partial Least Squares with Wide Kernel Algorithm (PLS), Principal Component Regression Algorithm (PCR) and Support Vector Machine (SVM) (Fig 3C).

Some models were possibly overfitted (Bayesian Naive Neural Network [BRNN], Random Forest [RF], TreeBag and xbgDART [XGB]), performing very well in the first part of data analysis (low RMSE values) but inconsistently in the cross validation process (Fig 3B). The K-Nearest Neighbors Algorithm (KNN model), presenting a good RMSE, underwent the underfitting phenomenon obtaining practically the same prediction for all 399 municipalities with no real variability for each one (Fig 3A).

Sensitivity tests were performed to validate and decide the performance of the models based on the validation criteria and consistency of the prediction errors obtained by the best performed models (SVM and PCR). Simulations were performed to confirm the results and observe the robustness of the outcomes in each model and to evaluate the error variation range.

**Fig 4:**
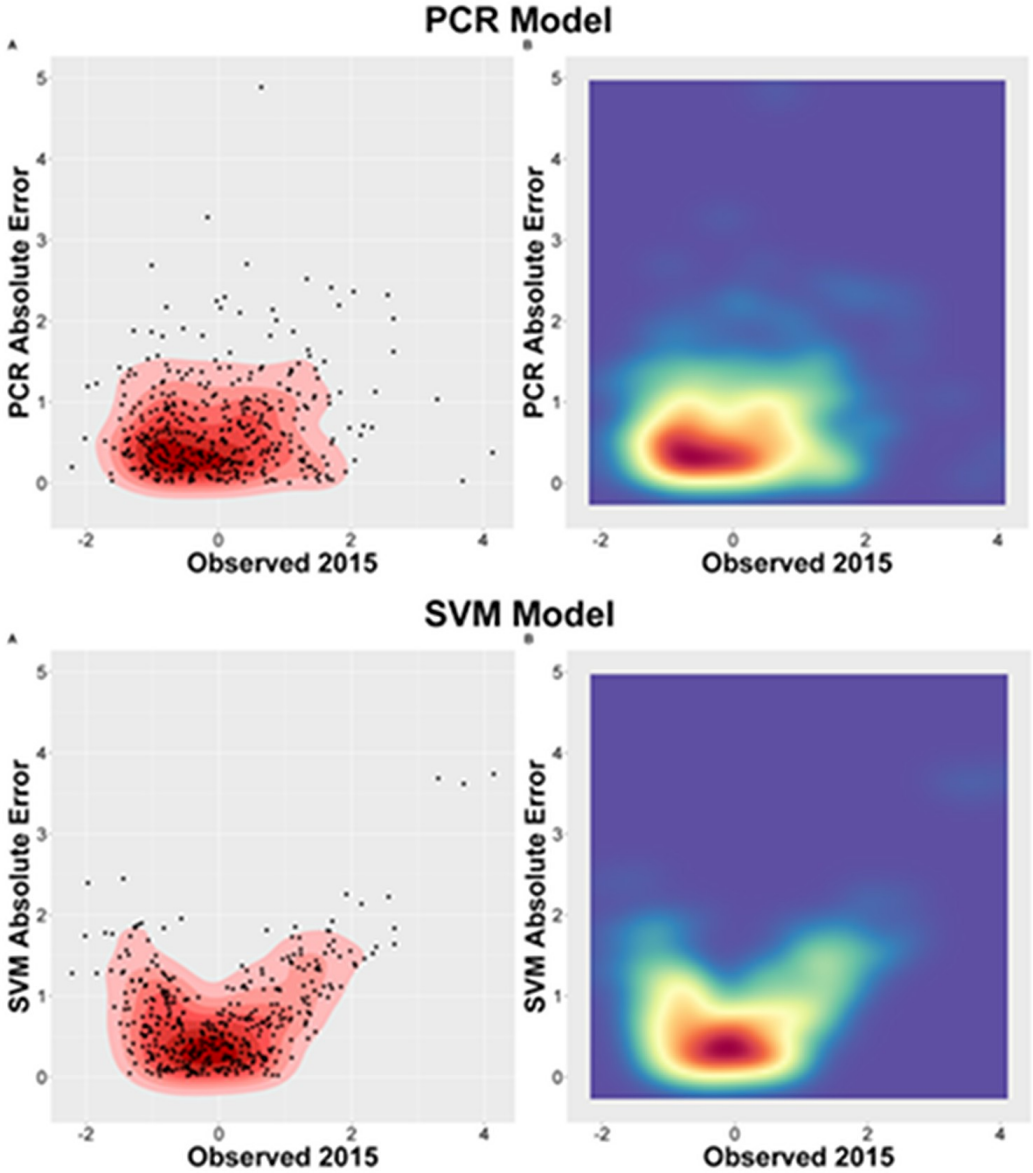
RMSE distribution representation. A—PCR model (2015) B—SVM model (2015).

The prediction error presented different distribution patterns based on the observed mortality rate value, even though the mean error (RMSE) was approximately identical in both models. The SVM model presented smallest errors concentration when the mortality rate approached its mean value and increasing errors as it approached the rate limits. In contrast, the PCR model presented a less homogeneous distribution pattern of its errors. The variation showed greater independence in relation to the predicted value and did not demonstrate a clear correlation with the observed value, being able to be an outcome of prediction that generalizes predicted values. To demonstrate the difference between the models, a density plot was generated (Fig 4). The different distributions of the error in both models are reflections of the predictive mechanism of each algorithm.

The weight for the SVM model variables are presented on S1 Fig. The top five positive related variables were aging rate, illiteracy, family health strategy coverage ratio, rates of consultation for diabetic population and municipal classification (small [regular]) and the top five negative related variables were advanced ambulances, GINI index, spatial accessibility index: hemodynamic laboratories, myocardial scintigraphy and basic ambulances.

### Model validation

The Support Vector Machine model was subjected to 30 repetitions of train-testing and prediction on 2015 data. The SVM model scored an average RMSE of 0.7904 with standard error of 0.0004 and ranging from 0.7901 to 0.7915. The best tune length value was determined to be 10. The C-value, a specific parameter for SVM models, was determined by the model through repetition of random values and selection of the one that produced the smallest RMSE in observed versus predicted values. In the lowest RMSE produced through repetition, C-values equaled 0.3694.

### Heart health care index

The SVM model predicted mortality rate was transformed into a standardized score of 0 to 1 where 1 is lower risk and 0 a higher risk. The municipalities were classified according to the risk of IHD mortality.

**Fig 5:**
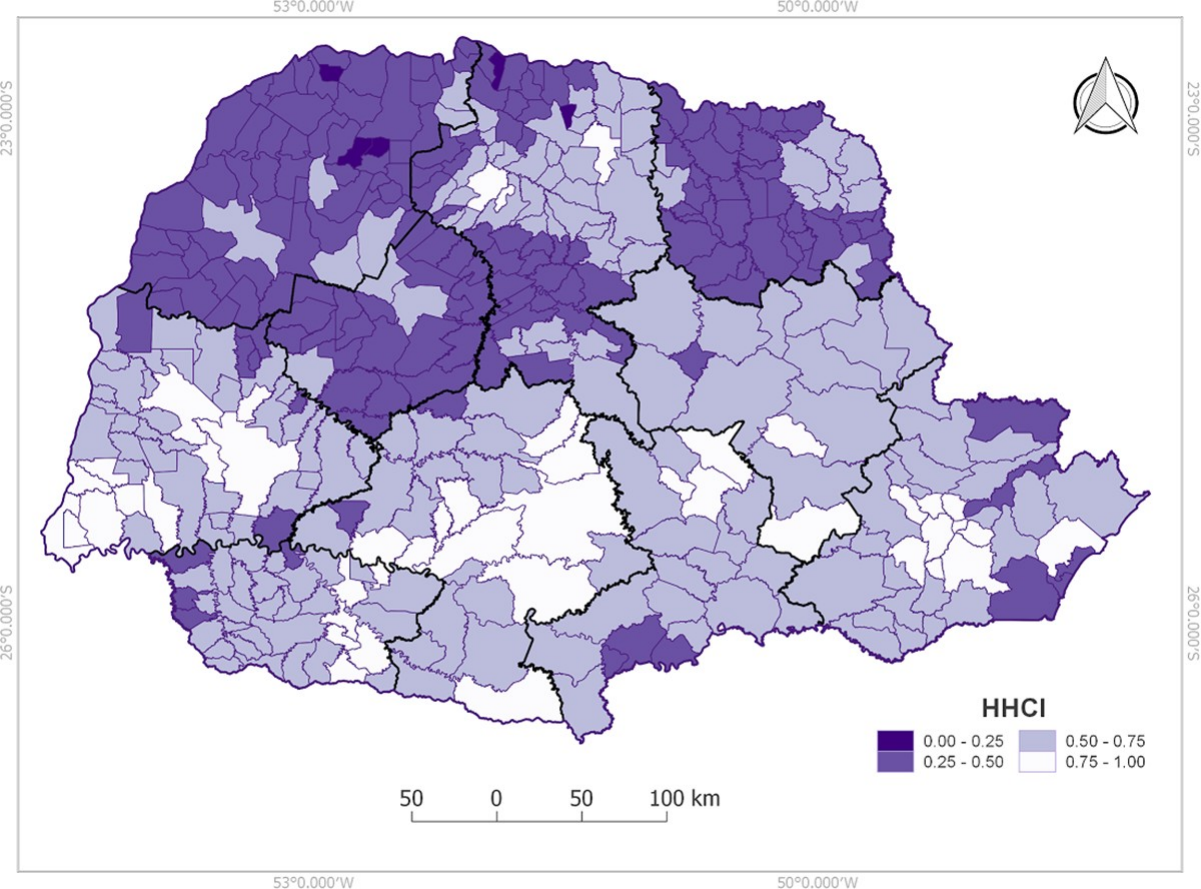
Distribution of the generated municipalities index. 1 lower risk and 0 higher risk.

Spatial distribution of the scores was plotted in a map showing the lowest indexes (greater risks) are in Northwest, Western Center, Pioneer North (large part) and Central North (South part) (Fig 5).

## Discussion

This was the first study to predict the IHD mortality rate in a Brazilian state´s municipalities considering socioeconomic, demographic and health accessibility indicators using machine learning to generate a heart health care index.

The variables aging rate, illiteracy, hemodynamic accessibility, IHD morbidity rate and advanced ambulances were the most important variables for HHCI formation showing how these factors may impact municipal IHD mortality.

Among the eight different decisions that machine learning models tested, three were seen with great potential for IHD mortality prediction. The SVM represented the lowest RMSE maintaining proportionality close to one [37] and more homogeneous concentration of errors showing higher correlation between observed and predicted model. A supervised model was chosen, having less learning independence but presenting an easier results interpretation. This model is widely used to make predictions [13] and allows the weights extraction of each variable to generate the index.

Aging rate was expected to be related to IHD mortality. However, illiteracy and accessibility indexes that are often discussed in scientific literature but not specifying its real importance and weight [3,7,8] were important for index formation and can be considered in public policies to reduce mortality. Examples are increasing the number of ambulances as well as the differential management of patients who are more than 120 minutes from the reference hemodynamics centers.

Health indicators weights directs actions to reduce heart health disparities, creating imperative strategies on public health education and awareness besides aging healthily [38–40] that is a big challenge for public managers when small and rural cities are part of the overall picture [41]. Currently, smaller and rural municipalities have less accessibility and longer waiting time after knowing a cardiac event has occurred, time to ambulance arrival and relocation of this individual to a reference hemodynamic center, generating higher risks of morbidity and mortality.

Only seven from ten administrative regions have an interventional cardiology center. North Paranaense is the exception; for all others, high-high IHD mortality clusters were observed where there are no high complexity interventional cardiology centers or at places further from the reference center corroborating previous findings about distance directly related to IHD mortality rates [6,42,43].

HHCI shows that the higher the mortality clusters, the lower the accessibility index. This is confirmed when the inverse is observed in the southern region where lower mortality rates have HHCI close to one indicating better accessibility. Thus, the HHCI and the main identified variables associated with IHD mortality rates may guide the municipalities’ health policy managers to improve such specific indicators to reduce population IHD mortality risk, followed by new cycles of evaluation, applying scientific prediction tools to health policy decisions.

Despite the presence of population heterogeneity, since the variables utilized were at municipal level, each municipal population presents specific singularities since each municipality is independently administered and presents specific general socioeconomic and healthcare realities, organization and policies. Thus, our approach is not supposed to be used on the individual level but rather be used as a municipality marker for health care regarding IHD, suitable to support municipal level government policies discussions and restructurations.

Thus, this study can be replicated and tested with socioeconomic, demographic and health coverage variables in different parts of the world, since the data are freely available. Machine learning allows the generation of results reliable to each region according to its characteristics, without incorrect generalizations or assumptions. With this methodology and secondary data, it is possible to have a municipal and state overview that can be monitored and re-evaluated periodically to verify improvements after a proposed plan’s execution.

Data availability for the 399 municipalities of the state with free access stands out as a strong point of the study, with no need to impute data.

One of the limitations of the study was the lack of some socioeconomic variable updating; however, the data from the last census provided by Brazilian Institute of Geography and Statistics (IBGE) remains very relevant. Nonetheless, to reduce this limitation, a greater number of variables available annually have been included and accessibility indexes were generated. Another limitation is the impossibility of generalization of the index because the data is only from one particular Brazilian state. Future research with more states or the entire Brazilian territory is necessary for a greater data volume and variability, making possible the generalization of the score.

These well-adjusted models need to be validated in other regions to the long-term objective of reducing the IHD mortality rates and having a municipal evaluation of them. Identifying and recognizing the main municipal determinants impacting health and predicting IHD mortality throughout the country will allow joint and guided decisions in health management to reduce morbidity and mortality rates as well as the generation of a care index which can assess heart health for each city in Brazil.

## Conclusion

Machine learning and big data in health area has grown and being accepted. By predicting the IHD mortality risk at the municipal level through the HHCI with freely obtained data, it becomes easier to generalize the model to the country and thus encourage decisions of health managers. This study showed how public data can and should be used for the development of projects that bring interventional results for health improvement.

## Supporting information

S1 Fig SVM model. Variables weight presentation.
(TIF)
