# Supplementary figures and images for: Mapping risk of ischemic heart disease using machine learning in a Brazilian state
Source: PLoS One. 2020 Dec 10;15(12):e0243558. doi: 10.1371/journal.pone.0243558 (PMC7728276; doi:10.1371/journal.pone.0243558)

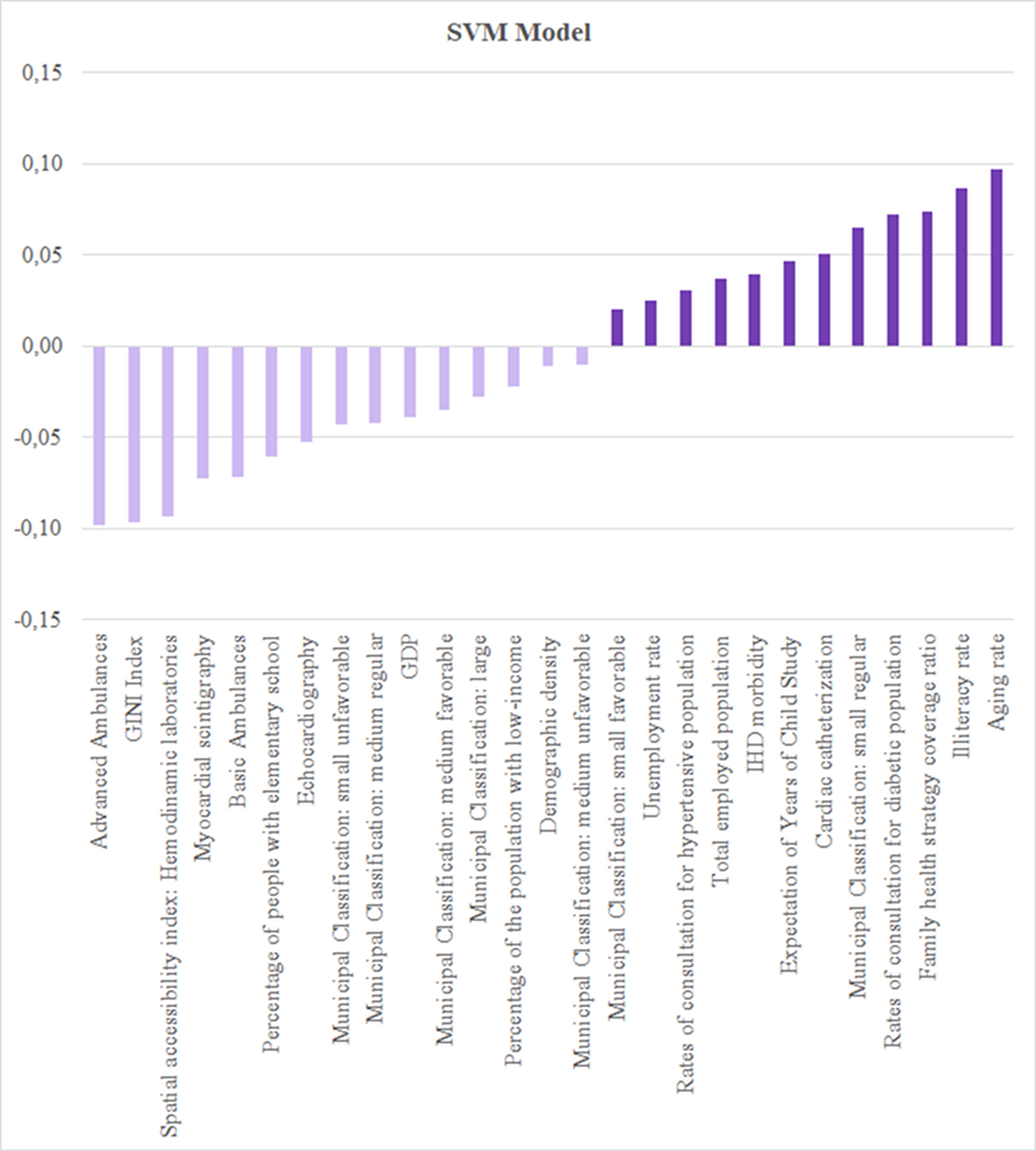

Supplement: S1 Fig — Variables weight presentation. (TIF) [file pone.0243558.s001.tif]
